# Left subclavian artery-esophageal fistula induced by a paper star: a case report

**DOI:** 10.7603/s40681-016-0018-0

**Published:** 2016-08-12

**Authors:** Chen-Sheng Lin, Cheng-Wen Lin

**Affiliations:** 1 Division of Gastroenterology, Kuang Tien General Hospital, 433 Taichung, Taiwan; 2 Department of Medical Laboratory Science and Biotechnology, China Medical University, No. 91, Hsueh-Shih Road, 404 Taichung, Taiwan; 3 Department of Biotechnology, Asia University, 413 Wufeng, Taichung, Taiwan

**Keywords:** Subclavian arteryesophageal fistula, Gastrointestinal bleeding, Hemorrhagic shock

## Abstract

A subclavian artery-esophageal fistula usually occurs on the right side of an aberrant subclavian artery. It also rarely appears in the site between a non-aberrant subclavian artery and the esophagus due to the ingestion of a foreign body. Upper gastrointestinal bleeding in the case of a subclavian artery-esophageal fistula is rare but often fatal. Here, we report on a 62-year-old male patient with a left subclavian arteryesophageal fistula complicated by hemorrhagic shock. He swallowed a foreign body at a birthday party. An upper gastrointestinal endoscopy indicated a paper star lodged at 20 cm from the incisors, inducing a kissing esophageal ulcer around the esophageal sphincter. One month later, he suffered an unusually strong episode of hematemesis. Subsequently, a computed tomography angiography was performed and demonstrated a left subclavian artery-esophageal fistula. Finally, the fistula induced by the ingestion of a paper star was successfully treated by endovascular stent grafting.

## 1. Introduction

A subclavian artery-esophageal fistula is rarely reported, but it can appear in life-threatening conditions. The aberrant right subclavian artery is the major clinical feature in subclavian arteryesophageal fistula and is found in between 0.5% to 1.8% of the general population [1]. Over 80% of cases diagnose the location as posterior to the esophagus, resulting in a high susceptibility to extrinsic compression and pressure necrosis [2, 3]. Therefore, long-term placement of nasogastric and endotracheal tubes become major risk factors to consider [2, 3].

Only a few cases of left subclavian artery-esophageal fistula have been reported, and these cases have generally been associated with the ingestion of a foreign body. Thus, cases with upper gastrointestinal bleeding and a history of foreign body ingestion should be under considerable suspicion for the possibility of a left subclavian artery-esophageal fistula.

## 2. Case report

**Figure 1:**
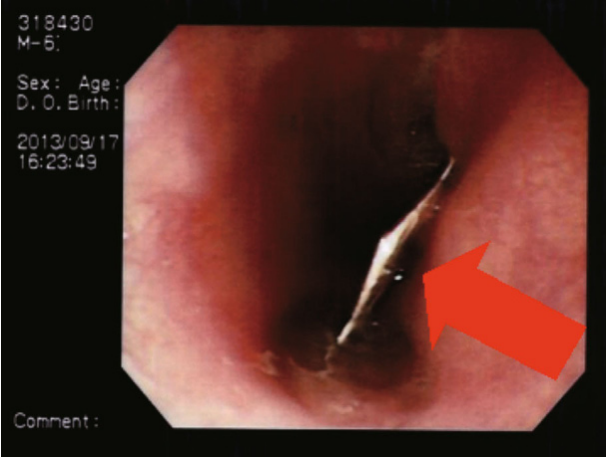

A 62-year-old male was admitted to the emergency department because he presented an episode of hematemesis after dinner. He had a history of left hemiparesis and language apraxia due to sequelae of cerebrovascular disease 3 years previously. He had also just been discharged from our internal medicine ward because of severe community-acquired pneumonia, left lower lobe, one month prior. His hemoglobin dropped to 5.8 mg/dl on the fifth day of this admission. An upper gastrointestinal endoscopy revealed a paper star lodged at 20 cm from the incisors (Fig. 1). A deep, kissing esophageal ulcer around the esophageal sphincter was found.

Initial vital signs at the emergency department showed a temperature of 36.5°C, a heart rate of 111 beats/minute, blood pressure of 146/111 mmHg, and a respiratory rate of 22 breaths/min. A physical examination revealed an acute, ill-looking elderly man who appeared uncomfortable and slightly disoriented. Volume resuscitation with crystalloid intravenous fluids was initiated. A blood transfusion with 2 units of packed red blood cells was given. A chest X-ray revealed mild atherosclerotic change and tortuous of aorta. Laboratory findings showed a white blood cell count of 17,290/ml with 59% neutrophils, 35% lymphocytes, a hemoglobin level of 9.0 g/dl, a platelet count of 224,000/ml, blood urea nitrogen of 21 mg/dl, creatinine of 1.06 mg/dl, sodium of 133mmol/L, and potassium of 3.3mmol/L.

**Figure 2:**
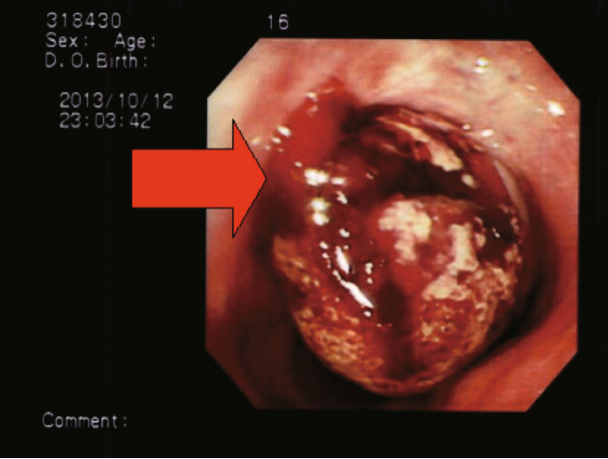

**Figure 3:**
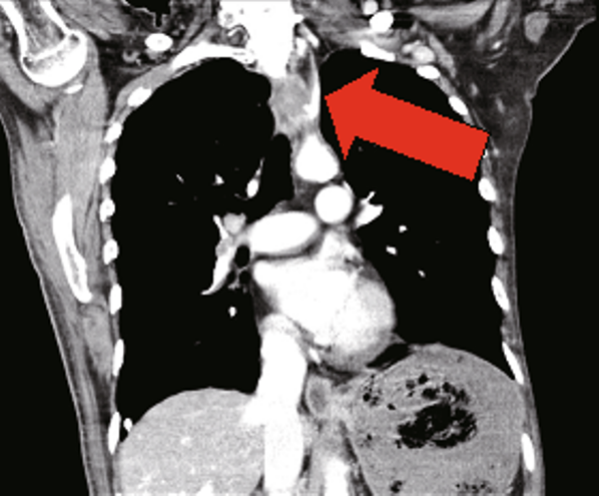

Owing to subsequent hypotension and massive hematemesis, an endotracheal intubation for airway protection was done in the ICU. An urgent upper gastrointestinal endoscopy revealed much fresh blood and a hematoma within the stomach, and one oval shaped blood clot that coated the upper esophagus (Fig. 2). Subsequently, a left subclavian artery-esophageal fistula was found by computed tomography angiography (Fig. 3).

**Figure 4:**
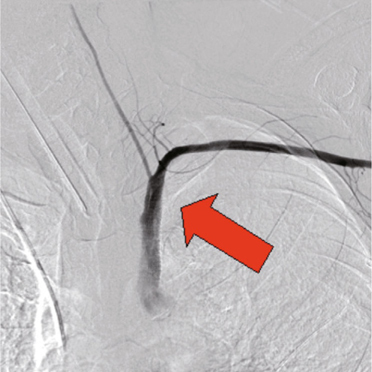

An emergency endovascular intervention was planned. An angiography showed a left subclavian artery to esophagus fistula with a rupture, and then one 11 mm × 5 cm Viabann stent graft was placed (Fig. 4). After the operation, no endoleak was noted. Feeding jejunostomy was performed for nutrition support. The patient was then discharged with signs of improvement one month later.

## 3. Discussion

A fistula between a non-aberrant subclavian artery and the esophagus is generally associated with the ingestion of a foreign body (ex. chicken bone or fish bone). It differs from an aberrant right subclavian artery fistula located posterior to the esophagus, which has a high susceptibility to extrinsic compression and pressure necrosis.

A subclavian artery-esophageal fistula should always be suspected in patients with the following conditions: (i) been placed long term with nasogastric and endotracheal tubes, with hematemesis; (ii) undergone aortic and/or esophageal surgery; and (iii) had a history of foreign body ingestion.

The clinical presentation of a subclavian artery-esophageal fistula resembles that of an aorto-esophageal fistula. Chiari first described the “aorto-esophageal syndrome” that includes the following series of symptoms: an event causing mid-thoracic pain, a sentinel arterial haemorrhage, a symptom-free period, and fatal exsanguinations [4].

As described in this case, the 62-year-old male with a history of cerebrovascular accidents occasionally choked if he swallowed too quickly without chewing thoroughly. Owing to language apraxia, it was hard for him to express discomfort after choking on a foreign body. He presented the typical Chiari triad: an ingestion of foreign body (a paper star) one month previously, one episode of vomiting blood, followed by one exsanguinating hematemesis.

While an upper gastrointestinal endoscopy is a good choice for diagnostic study, there still exist several complicating factors including an inability to identify a bleeding point due to the large volume of blood in the esophageal lumen and a small fistula caused by a foreign body. Therefore, the immediate insertion of a Sengstaken–Blakemore tube for exsanguination is the only method to temporarily buy time for a more definitive diagnosis to be made and management to be done.

Aortography is used for diagnosis when an aorto-esophageal fistula or subclavian artery-esophageal fistula is strongly suspected, but the use of aortography depends on local expertise. Furthermore, a computed tomography angiography with contrast extravasation from the subclavian artery into the esophagus can be highly suggestive in offering a rapid and definite method for diagnosing a fistula, showing esophageal perforation, a foreign body, a mediastinal abscess, and esophageal malignancy.

A thoracotomy with a vascular stent-graft interposition followed by esophageal surgery and mediastinal drainage is currently the most widely accepted approach for treating a subclavian artery-esophageal fistula. The unique feature about our case was that because it was treatment for a left subclavian arteryesophageal fistula with a vascular stent-graft we had to avoid an open surgical repair, since previous studies showed that such an approach caused extremely high mortality and morbidity rates [5].

In the end, the most important factor is early recognition. Early recognition is important for the management of any subclavian artery-esophageal fistula, and such early recognition depends on a high index of suspicion, improved critical care, as well as emergency surgical or endovascular interventions.
